# Development of Simultaneous Derivative Spectrophotometric and HPLC Methods for Determination of 17-Beta-Estradiol and Drospirenone in Combined Dosage Form

**DOI:** 10.1155/2015/534065

**Published:** 2015-04-27

**Authors:** Zeynep Aydoğmuş, Ece Merve Yılmaz, Sevgi Yörüsün, Samet Akpınar

**Affiliations:** Department of Analytical Chemistry, Faculty of Pharmacy, Istanbul University, Beyazıt, 34116 Istanbul, Turkey

## Abstract

Simple, rapid spectrophotometric, and reverse-phase high performance liquid chromatographic methods were developed for the concurrent analysis of 17-beta-estradiol (ESR) and drospirenone (DRS). The spectrophotometric method was based on the determination of first derivative spectra and determined ESR and DRS using the zero-crossing technique at 208 and 282 nm, respectively, in methanol. The linear range was 0.5–32.0 *µ*g·mL^−1^ for DRS and 0.5–8.0 *µ*g·mL^−1^ for EST. The limit of detection (LOD) values were 0.14 *µ*g·mL^−1^ and 0.10 *µ*g·mL^−1^ and limit of quantification (LOQ) values were 0.42 *µ*g·mL^−1^ and 0.29 *µ*g·mL^−1^ for ESR and DRS, respectively. The chromatographic method was based on the separation of both analytes on a C_18_ column with a mobile phase containing acetonitrile and water (70 : 30, v/v). Detection was performed with a UV-photodiode array detector at 279 nm. The linear range was 0.08–2.5 *µ*g·mL^−1^ for DRS and 0.23–7.5 *µ*g·mL^−1^ for EST. LOD values were 0.05 *µ*g·mL^−1^ and 0.02 *µ*g·mL^−1^ and LOQ values were 0.15 *µ*g·mL^−1^ and 0.05 *µ*g·mL^−1^ for ESR and DRS, respectively. These recommended methods have been applied for the simultaneous determination of ESR and DRS in their tablets.

## 1. Introduction

**Figure 1:**
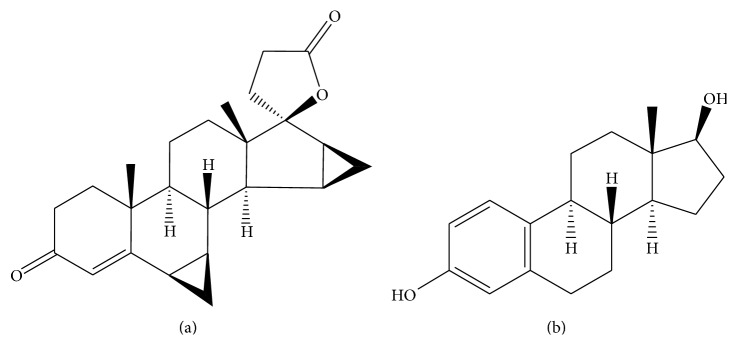
Molecular structure of drospirenone (a) and 17*β*-estradiol (b).

Drospirenone (DRS), chemically (6R,7R,8R,9S,10R,13S,14S,15S,16S,17S) 1,3′,4′,6,6a,7,8,9,10,11,12,13,14,15,15a,16-hexadecahydro-10, 13-dimethylspiro-[17H-dicyclopropa [6,7:15,16]cyclopenta[a]phenanthrene-17, 2′(5′H)-furan]-3,5′(2H)-dione (Figure 1), is used in contraception and hormone replacement therapy after menopause [1, 2].

17*β*-estradiol (ESR), chemically (17*β*)-estra-1,3,5(10)-triene-3,17-diol (Figure 1), is the most potent form in mammalian estrogenic steroids. It is firstly produced by the ovaries and is used in postmenopausal estrogen deprivation. The combination of drospirenone and 17-*β*-estradiol is used to treat menopause symptoms [1, 2].

So far, some high performance liquid chromatograph (HPLC) techniques coupled with ultraviolet (UV) [3–5], radioimmunoassay (RIA) [1, 2], and tandem mass spectrometry (MS/MS) methods [6, 7] have been published for quantification and pharmacokinetic studies of drospirenone alone and in combination with drugs in pharmaceutical formulations [4–6] and biological fluids [1, 2, 6, 7].

A number of efficient analytical techniques and procedures have been developed for the determination and pharmacokinetic studies of ESR individually as well as in combination with other drugs in pharmaceutical formulations, biological matrices, nutrients, and in* water* from different sources. For the determination of ESR, HPLC coupled with tandem mass spectrometry (MS/MS) [2, 8–10] methods has been widely used since they are highly sensitive and efficient methods, particularly in biological matrices. However, the HPLC-MS/MS method is expensive to analyze and time consuming and requires complicated procedures. Some HPLC with fluorescence (Fl) detection methods [11–14] and gas chromatography coupled to MS methods [1] have also been applied for the determination of ESR. Although these methods are sensitive, derivatization is usually required. Various HPLC-UV detection techniques, which are used commonly for the separation of comparatively high concentrations of drugs, have been reported for the determination of ESR in combination with different related estrogenic compounds in pharmaceutical dosage forms [15, 16], biological matrices [17–22], nutrients [23–27], and* waters* [28–36].

To the best of our knowledge, the simultaneous determination of ESR and DRS with the HPLC-UV method and spectrophotometric method has not yet been reported in the literature. The purpose of this study was to develop and validate an easy, precise, and selective RP-HPLC and derivative spectrophotometric method for the simultaneous determination of drugs in bulk and in tablets.

## 2. Experimental

### 2.1. Apparatus and Conditions

Spectrophotometric measurements were carried out with a Shimadzu UV-160 double beam spectrophotometer. Analysis was performed on the following operating conditions: 1-cm path length quartz cells, high scan speed, scan range 200–400 nm, 2 nm of slits width, and derivatives interval (Δ*λ*) of 1 nm.

HPLC measurements were performed on the Thermo Separation system (San Jose, CA) with the following parts: controller SN 4000, pump P 4000 and auto sampler AS 3000, fitted with 20 *μ*L sample loop, and photodiode array detector UV (UV-DAD) 6000 LP. Data acquisition was performed with ChromQuest 5.0 software.

Separation on a Waters Symmetry C_18_ column (4.6 mm × 250 mm, in diameter 5 *μ*m) was performed. The mobile phase of acetonitrile and water (70 : 30) was used with an isocratic mode at ambient temperature, 1 mL/min flow rate. The eluents were monitored at *λ* = 279 nm for both compounds. The mobile phase was filtered through a 0.45 *μ*m HV filter with a Millipore vacuum filter system. The pure water was obtained by an aquaMAXTM-ultra water purification device (Young-lin instrument, South Korea).

### 2.2. Materials and Solutions

Drospirenone (DRS) and 17*β*-estradiol (EST) were obtained from Sigma Aldrich. All solvents and chemicals with HPLC grade were purchased from Merck. Angeliq tablets were purchased from a local pharmacy.

Stock solutions of the studied drugs at 1.0 mg·mL^−1^ were prepared separately in methanol. The preparations of working standard solutions were made by appropriate dilutions from stock solution in methanol for the spectrophotometric methods and with acetonitrile-water (70 : 30, v/v) for the HPLC method.

### 2.3. General Procedures and Calibration Curves

#### 2.3.1. Derivative Spectrophotometric Method

Aliquots of standard solution of ESR and DRS (each 0.1 mg·mL^−1^) in mixture were transferred into 10 mL volumetric flasks to obtain the final concentrations of 0.5–8 *μ*g mL^−1^ for ESR and 0.5–32 *μ*g·mL^−1^ for DRS in methanol.

The zero order and first order derivative absorption spectra of standard solutions in the range of 200–400 nm were recorded against a blank solvent. Firstly, the zero order spectra were recorded and then they transformed into their first derivative order form. Zero-crossing amplitudes in the first order derivative spectra were measured at 208 and 282 nm for ESR and DRS, respectively. Each concentration level was performed using 6 independent assays. To determine the calibration curves, the first order derivative amplitude values of each compound were plotted against the concentrations and the corresponding regression equations were obtained.

#### 2.3.2. HPLC Method

The standard solutions of ESR and DRS in the mixture at six different concentration levels were transferred into 10 mL volumetric flasks to achieve final concentrations of 0.23–7.5 *μ*g·mL^−1^ for ESR and 0.08–2.5 *μ*g·mL^−1^ for DRS in the mobile phase and injected into the HPLC system. Six replicates for each concentration level were performed. The peak areas plotted against the concentration of the compounds under the optimized conditions to obtain calibration curves and the corresponding regression equations were obtained.

### 2.4. Determination of Drug in Tablets

Five tablets were weighed and finely powdered. The powder equivalent to an average tablet was weighed and then transferred to a 50 mL volumetric flask with 30 mL methanol and sonicated at room temperature for 1 h. The volume was completed with methanol and filtered. Tablet solution was appropriately diluted with methanol for the derivative spectrophotometric method and with acetonitrile : water (70 : 30, v/v) for the HPLC method. The solutions were then determined under specified conditions as in the section “general procedures and calibration curves.” Corresponding amounts of the drugs in the tablets were analyzed by related regression equations of the calibration curves.

## 3. Results and Discussion

### 3.1. Development of the First Derivative UV Spectroscopic Method

**Figure 2:**
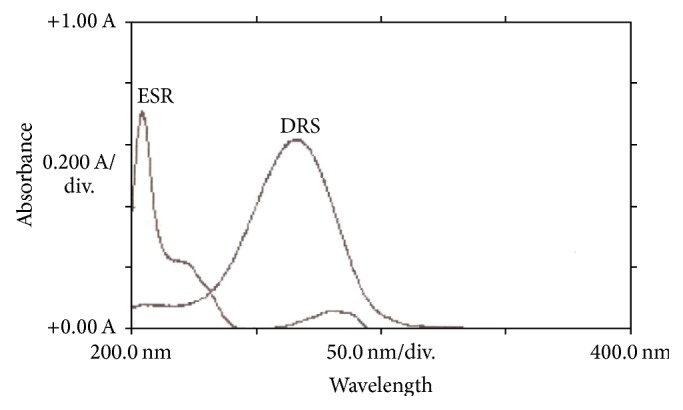
Absorption spectra of ESR and DRS in methanol (both are 5 *μ*g/mL).

Direct UV-absorption method was found to be inappropriate for the simultaneous determination of ESR and DRS due to some spectral interference. In addition, the wavelength of absorbance of ESR was lower than 205 nm and gave absorption bonds that were not sharp enough especially at low concentrations (Figure 2).

However, derivative spectrophotometry which is based on mathematical transformation has the advantages of reducing background absorbance and increasing the resolution of overlapping spectral bands and allows for the simultaneous analysis of organic compounds in the mixtures. Other important advantages of derivative spectroscopy are suppressing broad bands relatively to sharp bands and developing spectral details.

**Figure 3:**
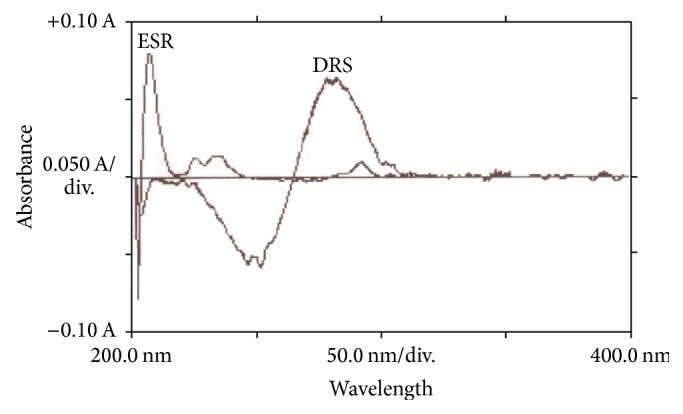
First order derivative absorption spectra of ESR (*λ*_max⁡_ = 208 nm) and DRS (*λ*_max⁡_ = 282 nm) in methanol (both are 5 *μ*g/mL).

For the reasons described hereinabove, the derivative spectra of ESR and DRS solutions from first up to fourth were recorded separately and their spectra were compared in a row by memory of the device. The 1st order derivative (1D) spectroscopy was chosen for simultaneous determination due to the obtained zero crossing points for both compounds. The optimum wavelength without interferences for EST and DRS was 208 and 282 nm, respectively (Figure 3).

For the derivative UV spectrophotometric method, methanol and acetonitrile alone and with mixtures of 50% water were tested as the solvent and methanol was found to be the most suitable solvent by considering the sensitivity, noise level, and resolution.

### 3.2. Development of the HPLC Method

**Figure 4:**
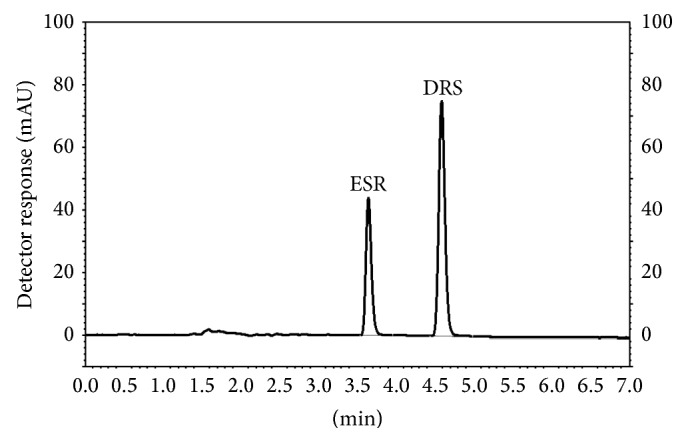
Schematic representation of chromatogram of ESR and DRS in selected conditions (both are 1.70 *μ*g/mL).

An RP-HPLC method has also been developed for the simultaneous determination of ESR and DRS. In order to improve the resolution of the drugs, methanol-water and acetonitrile-water in different portions were tested as the mobile phase. The best results in terms of obtaining sharp peaks, resolution, and analysis time were obtained using acetonitrile : water (70 : 30, v/v). A Phenomenex C_18_-column, Venusil XBP C_18_ (Agela), and a Waters Symmetry C_18_-column were tried to obtain the best separation. Waters Symmetry C_18_-column was selected for the accurate quantitation of both drugs. The optimized detection wavelengths and flow rate were 279 nm and 1 mL/min, respectively, at room temperature. The average retention time of the ESR and DRS was approximately 3.54 and 4.55 min, respectively. RSD% of the retention times for both drugs was approximately 2.18% for 9 independent analyses. A typical chromatogram of drugs in mixture in selected conditions is shown in Figure 4.

### 3.3. Method Validation

#### 3.3.1. Linearity and Sensitivity

Calibration curves parameters were summarized in Table 1. For the derivative spectrometry method, the linearity range of ESR and DRS was found as 0.5–8.0 *μ*g·mL^−1^ and 0.5–32 *μ*g·mL^−1^, respectively. For the HPLC method, the linearity range of ESR and DRS was found as 0.23–7.5 *μ*g·mL^−1^ and 0.08–2.5 *μ*g·mL^−1^, respectively. In both cases, correlation coefficients (*r* ^2^) were greater than 0.9967, indicating good linearity (Table 1).

Limit of detection (LOD) and quantification (LOQ) of drugs for proposed methods was calculated with the following equation: LOD = 3.3 *S*_*a*_/*b* and LOQ = 10 *S*_*a*_/*b*, where *S*_*a*_ is the standard deviation of the intercept and *b* is the slope of calibration curve [37]. LOD and LOQ values were 0.14 and 0.42 *μ*g·mL^−1^ for ESR, 0.10, and 0.29 *μ*g·mL^−1^ for DRS, respectively, for first derivative spectrometry method. The LOD and LOQ values were 0.05 and 0.15 *μ*g·mL^−1^ for ESR, 0.02, and 0.05 *μ*g·mL^−1^ for DRS, respectively, for the HPLC method (Table 1).

#### 3.3.2. Accuracy and Precision

Intraday and interday accuracy and precision were validated by solutions of drugs at three different concentrations for both proposed methods. Determinations were performed at five replicates within the same day for intraday and on five separate days for interday precision. For intraday and interday precision, the percent relative standard deviation (RSD%) values of ESR ranged from 0.01 to 0.32% and 0.45 to 1.07%, respectively, for the derivative spectroscopy method (Table 2) and 0.02 to 1.18% and 0.08 to 1.03%, respectively, for the HPLC method (Table 2). RSD% values of DRS ranged from 1.04 to 1.94% and 1.17 to 2.84%, respectively, for spectrometry and 0.69 to 1.90% and 0.15 to 2.67%, respectively, for the HPLC method.

#### 3.3.3. Recovery

Recovery studies were conducted by spiking known amounts of pure compounds solutions at three different concentrations to a known amount of tablet solutions.

The results given in Table 3 revealed that the percent recovery for ESR by derivative spectrophotometry and HPLC methods was in the range of 91.75–104.62% and 98.75–106.57%, respectively. The recovery values for DRS were 96.40–100.00% and 93.33–96.50% for the derivative spectrophotometric and HPLC methods, respectively. The recovery results offer that the method is not affected by the presence of the excipients in the formulation and confirms the high accuracy. The RSD% values of both drugs for both methods were less than 7.72% (Table 3).

#### 3.3.4. Stability and Specificity

To examine the stability of the ESR and DRS solutions, the compounds in the mixture stored in the refrigerator at +4°C for a month and in the dark for 4 days at room temperature and then were analyzed in three replicates by the proposed methods under the selected conditions. The analyses results of these samples were compared with the results of freshly prepared drug solutions and found to be stable under these conditions.

### 3.4. Application to Tablets

The proposed methods were administered for the analysis of the drugs studied in their tablet form, namely, Angeliq, which contains 1 mg ESR and 2 mg DRS per tablet. For the first derivative spectrophotometric method, the mean recovery values were 101% (RSD% = 0.79) and 99% (RSD% = 0.03) for ESR and DRS, respectively (Table 4). For the HPLC method, the mean recovery values were 97% (RSD% = 0.21) and 98% (RSD% = 0.36) for ESR and DRS, respectively (Table 4).

## 4. Conclusion

In this study, a simple, rapid, accurate, and sensitive first derivative spectrophotometric and an RP-HPLC method were developed and validated for the simultaneous determination of ESR and DRS in their tablets for the first time. The HPLC method has a shorter analytical run. Both methods are cost effective compared to the LC-MS methods. Considering the linearity values and LOD values of DRS, the both proposed methods were more sensitive than reported RP-HPLC methods for the assay of the drug alone [5] and in combination with ethynyl estradiol in pharmaceutical preparations [3, 4]. The proposed methods for the determination of ESR were found to be more sensitive than some of the published HPLC-UV methods [16, 22, 33]. In addition, the LOD value of ESR for the proposed RP-HPLC method was found to be more sensitive than the other reported HPLC methods [22, 25, 31, 33, 35, 36] and a spectrophotometric method that has been published very recently [38]. The methods developed can be successfully used in the laboratories of quality control for the routine analysis of both compounds in pure form and pharmaceutical forms without preseparation.

## Figures and Tables

**Table 1:** Results of analytical parameters of proposed methods.

Parameters	Derivative spectrophotometric method		HPLC method	
	ESR	DRS	ESR	DRS
Linearity range (*µ*g mL^−1^) (*n* = 6)	0.5–8.0	0.5–32.0	0.23–7.5	0.08–2.5
Regression equation^a^				
Slope	0.014	0.003	45.83	264.95
Intercept	0.009	0.001	1.23	2.208
Correlation coefficient (*r* ^2^)	0.9967	0.9998	0.9999	1.0
SD of *a*	0.000	0.000	0.842	2.86
SD of *b*	0.000	0.000	0.71	1.43
LOD (*µ*g mL^−1^)	0.14	0.10	0.05	0.02
LOQ (*µ*g mL^−1^)	0.42	0.29	0.15	0.05

^a^ *y* = *aC* + *b* (*C *is the concentration of drug in *µ*g mL^−1^ for both methods, *y *is absorbance at *λ*_max_ for derivative spectrophotometric method and peak area for HPLC method, *a* is slope, and *b* is intercept) and average of five and six determination points for spectrophotometric and HPLC method, respectively.

**Table 2:** The intraday and interday accuracy and precision analysis of ESR and DRS by derivative spectrophotometric and HPLC methods (*n* = 5).

Method	Drug	Concentration (*µ*g mL^−1^)	Intraday Found ± RSD, %	Interday Found ± RSD, %
Derivative spectrophotometric method	ESR	0.63	0.63 ± 0.32	0.56 ± 1.07
		2.50	2.44 ± 0.01	2.38 ± 0.50
		5.00	5.06 ± 0.01	4.94 ± 0.45
	DRS	1.00	0.89 ± 1.35	0.88 ± 2.39
		5.00	5.01 ± 1.94	4.93 ± 2.84
		16.00	16.31 ± 1.04	16.28 ± 1.17

HPLC method	ESR	0.63	0.62 ± 1.18	0.64 ± 1.03
		1.25	1.26 ± 0.21	1.26 ± 0.79
		5.00	5.00 ± 0.02	5.01 ± 0.08
	DRS	0.16	0.16 ± 0.69	0.15 ± 2.67
		0.63	0.63 ± 1.90	0.62 ± 2.58
		1.25	1.26 ± 1.75	1.24 ± 0.15

**Table 3:** Recovery of ESR and DRS determined by the proposed methods (*n* = 6).

Method	Concentration (*µ*g mL^−1^)			Recovery (%)	RSD (%)
	Taken	Added	Found ± SD		
Derivative spectrophotometric method					
ESR	1	0.3	1.36 ± 0.07	104.62	5.44
		1.5	2.59 ± 0.06	103.60	2.24
		3	3.67 ± 0.06	91.75	1.72
DRS	2	0.3	2.30 ± 0.02	100.00	0.70
		1.5	3.45 ± 0.16	98.57	4.55
		3	4.82 ± 0.17	96.40	3.52
HPLC method					
ESR	0.5	0.3	0.79 ± 0.06	98.75	7.72
		1.5	1.99 ± 0.04	99.50	1.96
		3	3.73 ± 0.05	106.57	1.23
DRS	1	0.2	1.12 ± 0.03	93.33	2.77
		0.7	1.63 ± 0.03	95.88	1.96
		1	1.93 ± 0.02	96.50	1.03

**Table 4:** Analysis of ESR and DRS in Angeliq tablets by developed methods (1 mg ESR and 2 mg DRS per tablet), *n* = 6.

Statistical values	Derivative spectrophotometric method	HPLC method	Derivative spectrophotometric method	HPLC method
	ESR		DRS	
Mean (mg) ± SD	1.01 ± 0.008	0.97 ± 0.002	1.98 ± 0.000	1.96 ± 0.007
Recovery (%)	101	97	99	98
RSD (%)	0.79	0.21	0.03	0.36

## References

[1] Sutter G., Schmelter T., Gude K., Schaefers M., Gerlinger C., Archer D. F. (2014). Population pharmacokinetic/pharmacodynamic evaluation of low-dose drospirenone with 17*β*-estradiol in postmenopausal women with moderate to severe vasomotor symptoms. *Menopause*.

[2] Karara A. H., Hanes V., Alonso A. (2007). Pharmacokinetics and pharmacodynamics of drospirenone-estradiol combination hormone therapy product coadministered with hydrochlorothiazide in hypertensive postmenopausal women. *Journal of Clinical Pharmacology*.

[3] Silva V. B., Galdos A. A. G., Mothe C. M. A. (2013). Simultaneous determination of ethinyl estradiol and drospirenone in oral contraceptive by high performance liquid chromatography. *Brazilian Journal of Pharmaceutical Sciences*.

[4] Patel R. C., Rathod D. K., Rajesh K. S., Patel V. S. (2013). RP-HPLC method development and validation for estimation of drospirenone and ethinyl estradiol in bulk and combined dosage form. *Pharmagene*.

[5] Pradad G. R., Srinivas B. P., Ramana M. V. (2011). Validated RP—HPLC method for the estimation of drospirenone in formulation. *International Journal of Research in Pharmaceutical and Biomedical Sciences*.

[6] Moser C., Zoderer D., Luef G. (2012). Simultaneous online SPE-LC-MS/MS quantification of six widely used synthetic progestins in human plasma. *Analytical and Bioanalytical Chemistry*.

[7] Bhaumik U., Ghosh A., Mandal U. (2008). Determination of drospirenone in human plasma by LC-tandem-MS. *Chromatographia*.

[8] Tai S. S.-C., Welch M. J. (2005). Development and evaluation of a reference measurement procedure for the determination of estradiol-17*β* in human serum using isotope-dilution liquid chromatography-tandem mass spectrometry. *Analytical Chemistry*.

[9] Guedes-Alonso R., Sosa-Ferrera Z., Santana-Rodríguez J. J. (2013). Simultaneous determination of hormonal residues in treated waters using ultrahigh performance liquid chromatography-tandem mass spectrometry. *Journal of Analytical Methods in Chemistry*.

[10] Gouveia M. J., Brindley P. J., Santos L. L., Correia da Costa J. M., Gomes P., Vale N. (2013). Mass spectrometry techniques in the survey of steroid metabolites as potential disease biomarkers: a review. *Metabolism: Clinical and Experimental*.

[11] Gatti R., Gioia M. G., Di Pietra A. M., Cavrini V. (1998). HPLC-fluorescence determination of unconjugated estrogens in pharmaceuticals. *Journal of Pharmaceutical and Biomedical Analysis*.

[12] Roos R. W. (1978). High-pressure liquid chromatographic analysis of estrogens in pharmaceuticals by measurement of their dansyl derivatives. *Journal of Pharmaceutical Sciences*.

[13] Yilmaz B., Kadioğlu Y. (2013). Quantitation of 17 *β*-estradiol in rabbit plasma by high-performance liquid chromatography with fluorescence detection. *Journal of Liquid Chromatography & Related Technologies*.

[14] Wen Y., Zhou B.-S., Xu Y., Jin S.-W., Feng Y.-Q. (2006). Analysis of estrogens in environmental waters using polymer monolith in-polyether ether ketone tube solid-phase microextraction combined with high-performance liquid chromatography. *Journal of Chromatography A*.

[15] Nováková L., Solich P., Matysová L., Šícha J. (2004). HPLC determination of estradiol, its degradation product, and preservatives in new topical formulation Estrogel HBF. *Analytical and Bioanalytical Chemistry*.

[16] Zhao S., Wu D., Wang P. (2004). Simultaneous determination of seven sexual hormones in cosmetics by reversed-phase high performance liquid chromatography. *Se Pu*.

[17] Formento J.-L., Moll J.-L., Francoual M. (1987). HPLC micromethod for simultnaeous measurement of estradiol, progesterone, androgen and glucocorticoid receptor levels. Application to breast cancer biopsies. *European Journal of Cancer and Clinical Oncology*.

[18] Suzuki Y., Hayashi N., Sekiba K. (1988). Automated direct assay system for the measurement of sex steroid hormones in serum using high-performance liquid chromatography. *Journal of Chromatography B: Biomedical Sciences and Applications*.

[19] Lamparczyk H., Zarzycki P. K., Nowakowska J., Ochocka R. J. (1994). Application of *β*-cyclodextrin for the analysis of estrogenic steroids in human urine by high-performance liquid chromatography. *Chromatographia*.

[20] Scherr F. F., Sarmah A. K. (2011). Simultaneous analysis of free and sulfo-conjugated steroid estrogens in artificial urine solution and agricultural soils by high-performance liquid chromatography. *Journal of Environmental Science and Health. Part B*.

[21] Zou Y., Li Y., Jin H. (2012). Determination of estrogens in human urine by high-performance liquid chromatography/diode array detection with ultrasound-assisted cloud-point extraction. *Analytical Biochemistry*.

[22] Chen B., Huang Y., He M., Hu B. (2013). Hollow fiber liquid-liquid-liquid microextraction combined with high performance liquid chromatography-ultraviolet detection for the determination of various environmental estrogens in environmental and biological samples. *Journal of Chromatography A*.

[23] Jiang T. H., Zhao L. X., Chu B. L., Feng Q. H., Yan W., Lin J. M. (2009). Molecularly imprinted solid-phase extraction for the selective determination of 17*β*-estradiol in fishery samples with high performance liquid chromatography. *Talanta*.

[24] Xiao X., Yin Y., Hu Y., Li G. (2010). Microwave-assisted extraction coupled with single drop micro extraction and high-performance column liquid chromatography for the determination of trace estrogen adulterants in soybean isoflavone dietary supplements. *Journal of AOAC International*.

[25] Hu Y., Wang Y., Chen X., Hu Y., Li G. (2010). A novel molecularly imprinted solid-phase microextraction fiber coupled with high performance liquid chromatography for analysis of trace estrogens in fishery samples. *Talanta*.

[26] Gañán J., Gallego-Picó A., Garcinuño R. M. (2012). Development of a molecularly imprinted polymer-matrix solid-phase dispersion method for selective determination of *β*-estradiol as anabolic growth promoter in goat milk. *Analytical and Bioanalytical Chemistry*.

[27] Socas-Rodríguez B., Asensio-Ramos M., Hernández-Borges J., Rodríguez-Delgado M. Á. (2013). Hollow-fiber liquid-phase micro extraction for the determination of natural and synthetic estrogens in milk samples. *Journal of Chromatography A*.

[28] Kitahara T., Takano J., Kitamir H., Watanabe T. (2002). Simultaneous determination of alkylphenols, bisphenol A and 17*β*-estradiol in environmental water by solid-phase extraction/HPLC. *Bunseki Kagaku*.

[29] Almeida C., Nogueira J. M. F. (2006). Determination of steroid sex hormones in water and urine matrices by stir bar sorptive extraction and liquid chromatography with diode array detection. *Journal of Pharmaceutical and Biomedical Analysis*.

[30] Stafiej A., Pyrzynska K., Regan F. (2007). Determination of anti-inflammatory drugs and estrogens in water by HPLC with UV detection. *Journal of Separation Science*.

[31] Wang C., Xu C., Chen F., Tang X. (2011). Simultaneous determination of three naturally occurring estrogens in environmental waters by high-performance liquid chromatography. *Journal of Separation Science*.

[32] Hadjmohammadi M. R., Ghoreishi S. S. (2011). Determination of estrogens in water samples using dispersive liquid liquid microextraction and high performance liquid chromatography. *Acta Chimica Slovenica*.

[33] Gañán J., Pérez-Quintanilla D., Morante-Zarcero S., Sierra I. (2013). Comparison of different mesoporous silicas for off-line solid phase extraction of 17*β*-estradiol from waters and its determination by HPLC-DAD. *Journal of Hazardous Materials*.

[34] Zou Y. M., Zhang Z., Shao X. L. (2014). Hollow-fiber-supported liquid-phase microextraction using an ionic liquid as the extractant for the pre-concentration of bisphenol A, 17-*β*-estradiol, estrone and diethylstilbestrol from water samples with HPLC detection. *Water Science and Technology*.

[35] Pérez R. L., Escandar G. M. (2014). Liquid chromatography with diode array detection and multivariate curve resolution for the selective and sensitive quantification of estrogens in natural waters. *Analytica Chimica Acta*.

[36] Cordeiro D., Da Rocha G. C., Onaka E. M. (2012). HPLC determination of hormones in São José do Rio Preto municipal DAM, São Paulo, Brazil. *Journal of Liquid Chromatography & Related Technologies*.

[37] ICH (2005). *ICH Guidelines Q2(R1), Harmonised Tripartite Guideline, Validation of Analytical Procedures: Text and Methodology*.

[38] Yilmaz B., Kadioglu Y. (2013). Determination of 17 *β*-estradiol in pharmaceutical preparation by UV spectrophotometry and high performance liquid chromatography methods. *Arabian Journal of Chemistry*.

